# Role of ambulation to prevent venous thromboembolism in medical patients: where do we stand?

**DOI:** 10.1590/1677-5449.180107

**Published:** 2019-06-25

**Authors:** Maria Chiara Chindamo, Marcos Arêas Marques

**Affiliations:** 1 Universidade Federal do Rio de Janeiro – UFRJ, Departamento de Clínica Médica, Rio de Janeiro, RJ, Brasil.; 2 Rede D’Or São Luiz, Hospital Barra D’Or, Serviço de Clínica Médica, Rio de Janeiro, RJ, Brasil.; 3 Universidade do Estado do Rio de Janeiro – UERJ, Hospital Universitário Pedro Ernesto, Rio de Janeiro, RJ, Brasil.; 4 Universidade Federal do Estado do Rio de Janeiro – UNIRIO, Hospital Universitário Gaffrée e Guinle, Rio de Janeiro, RJ, Brasil.

**Keywords:** deep venous thrombosis, pulmonary embolism, thromboprophylaxis, patient safety, mobility limitation

## Abstract

Venous thromboembolism (VTE) encompasses the spectrum of manifestations of deep venous thrombosis and/or pulmonary embolism and is a common, serious, and preventable complication in hospitalized patients. Although immobility plays an important role in determining VTE risk in medical patients, no clear and uniform criteria exist to guide clinicians in assessing immobility. The variation in the descriptions that do exist makes it difficult to interpret and compare the results of randomized clinical trials with respect to the influence of different levels of immobility on the magnitude of VTE risk and the role that early ambulation as an isolated factor plays in prevention of such events. Understanding these limitations is a prerequisite for the proper use and interpretation of VTE risk assessment tools and for indicating the best strategy for preventing venous thrombosis in hospitalized medical patients. The objective of this study was to review the main evidence reported in the literature on the role of ambulation in prevention of VTE.

## INTRODUCTION

Venous thromboembolism (VTE) is the greatest cause of avoidable deaths among hospitalized patients.^1^^,^ 2 Acquired and inherited risk factors involved in its genesis include obesity, prior thrombosis, thrombophilias, cancer, recent trauma or surgery, acute myocardial infarction, stroke, paresis or paralysis of lower limbs, advanced age, congestive heart failure, acute infection, hormone therapy, central venous catheters, and admission to intensive care units.^3^^-^ 6 Loss of mobility, whether recent or longstanding, is a common acquired risk factor that is important in development of VTE, increasing incidence by two to five times when compared to patients with normal mobility.^5^

Medical patients tend to have a history of restricted mobility caused by the acute disease that prompted their admission and also because of comorbidities. While immobility is one of the risk factors used to indicate VTE prophylaxis in hospitalized medical patients, pharmacological prophylaxis is still underutilized and return to walking is often the only criterion considered when deciding on withdrawal of thromboprophylaxis.^7^ Certain questions remain unanswered and this can make precise assessment of VTE risk difficult. Issues that are of particular importance include: a lack of consensus on the definitions of immobility used in large studies of thromboprophylaxis for medical patients, the degree to which immobility contributes to VTE risk, and the importance of early mobilization as a protective factor. The objective of this study was to review and evaluate the most important evidence in the medical literature related to the role of ambulation in VTE prophylaxis.

## REDUCED MOBILITY AND VTE RISK

Several authors have suggested that there is a relationship between reduced mobility and increased risk of VTE, proportional to the degree to which and length of time for which the patient is confined to bed.^8^^-^ 10 A prospective, multicenter, case-control study with hospitalized patients over the age of 65 years found that restriction of mobility was an independent risk factor for VTE (odds ratios: 1.73-5.64).^5^ Risk was greater among those with more severe mobility restriction (bedridden vs. wheelchair) and with recent loss of mobility (< 15 days vs. ≥ 30 days). Similarly, prolonged hospitalization has been identified as an independent risk factor for development of VTE, increasing the occurrence of events by up to eight times compared with patients who are not hospitalized.^11^

## DEFINITIONS OF IMMOBILITY

Different definitions have been employed in randomized clinical trials to describe immobility: 1) qualitative, described as a dichotomous variable (mobile or restricted mobility), or by levels (partial or total); or 2) quantitative, with descriptions of the time walked or the distance walked in 24 hours.^12^^,^ 13 Synonyms of immobility include restricted mobility, prolonged immobility, confinement to bed, or bed rest with bathroom privileges.^12^^,^ 13

Recently, Ye et al.^12^ published a systematic review of 21 randomized clinical trials highlighting the highly heterogeneous nature of definitions of immobility applied to hospitalized medical patients. Some definitions combine type and duration of immobility, as in the PRIME^14^ (immobilization expected for more than half of the day for a period of 7 days), PRINCE^15^ (bed confined for more than 2/3 of each day for 10±2 days), ARTEMIS^16^ (bed confined ≥ 4 days), PREVENT^17^ (projected hospitalization ≥ 4 days and ≤ 3 days of immobilization before hospitalization), and EXCLAIM studies^8^ (immobility classified in two levels: level 1, absolute bed rest or sedentary without bathroom privileges, and level 2, bed rest with bathroom privileges, considering ≥ 3 days projected hospital stay). The Prophylaxis of Venous Thromboembolism in MEDical Patients With ENOXaparin (MEDENOX) study^18^ employed quantitative parameters, defining immobility as an inability to walk > 10 meters unaided for a period of 10±4 days.

In that review, the authors concluded that, despite the established efficacy of VTE pharmacological prophylaxis in patients with acute clinical disease, there is still no consensus on the definition of immobility.^12^ The diversity of definitions and the predominantly qualitative descriptions make comparison of results problematic and make it impossible to precisely determine the levels or mobilization/ambulation that may contribute to reducing the incidence of VTE events.^19^^,^ 20

## IMMOBILITY AND VTE RISK STRATIFICATION MODELS IN CLINICAL PATIENTS

The best VTE risk stratification model for acutely ill medical patients has not yet been defined.^21^^-^ 23 Notwithstanding, these tools are very important for identifying patients who are eligible for pharmacological or mechanical prophylaxis while in hospital. Since rates of compliance with thromboprophylaxis protocols are low all over the world, many VTE risk stratification models have been developed to support clinical decision-making, improving use of pharmaceutical prophylaxis in at-risk populations.^21^^-^ 23

All validated stratification models include reduced mobility as a risk factor for VTE. The most recent update to the American College of Chest Physicians (ACCP) guidelines^3^ recommends using the Padua score as a VTE risk assessment tool in hospitalized medical patients. This model is made up of 11 risk factors, scored from one to three (with a maximum score of 20 points), for identifying clinical patients at high risk of VTE (score ≥ 4).^22^ Reduced mobility alone scores three points. A quantitative model that has also been validated externally is the International Medical Prevention Registry on Venous Thromboembolism (IMPROVE), which employs a wider-ranging definition of immobility.^23^ In Brazil, a group of specialists published the Brazilian VTE prophylaxis guidelines for hospitalized patients (Diretriz Brasileira de Profilaxia de TEV em Paciente Clínico Internado) in 2007, which precedes the scores described above. In these guidelines, age over 40 years and immobility combined with at least one other risk factor for VTE are sufficient grounds to prescribe pharmacological prophylaxis.^24^ Table 1 presents the definitions of immobility from these three risk assessment models.

**Table 1:** Definitions of immobility in VTE risk assessment models.

**Risk assessment models**	**Definition of immobility**	**Indications for pharmacological prophylaxis**
Padua score^22^	Probability of immobility because of limitations caused by disease or treatment or medical indications for at least 3 days	Immobility: 3 points. Pharmacological prophylaxis of benefit if score ≥ 4
IMPROVE^23^	Bed or chair rest > 24 hours for ≥ 7 days	Immobility: 1 point. Pharmacological prophylaxis of benefit if score ≥ 2
Brazilian Prophylaxis Guidelines for Hospitalized Clinical Patients^24^	Spends at least half of the day lying down or sitting on the edge of the bed (excluding time spent asleep) because of disease	Prophylaxis is indicated if immobility is present, age ≥ 40 years, and at least one risk factor is present

IMPROVE = *International Medical Prevention Registry on Venous Thromboembolism*.

## IMMOBILITY AND DETERMINATION OF PROPHYLAXIS DURATION

In clinical practice, the relationship between duration of VTE risk, duration of pharmacological prophylaxis, and early mobilization in hospitalized clinical patients remains a challenge. In addition to known limitations related to the divergent definitions of immobility, there are also no measurable standards for mobilization that can be correlated with reduction in VTE risk.

While the duration established for safe and effective prophylaxis in hospitalized clinical patients is from 6 to 14 days (mean of 7 days), as defined by the PREVENT, ARTEMIS, and MEDENOX studies,^16^^-^ 18 mobilization has often been used as the only criterion for withdrawal of prophylaxis.^7^ Considering the universal tendency to reduce the length of hospital stays, it has become unlikely in current practice that clinical patients will be given pharmacological prophylaxis for the recommended length of time while in hospital. Less than 2% of patients continue to receive pharmacological prophylaxis after hospital discharge, which causes an increase in the incidence of events out of hospital.^25^^,^ 26

In a real-life study, Amin et al.^26^ conducted a retrospective analysis of occurrence of symptomatic VTE within 180 days of admission in 11,139 patients with diagnoses of cancer, heart failure, severe lung disease, or infectious disease. The rate of VTE was 3.3%, after receiving pharmacological prophylaxis with a mean duration of 5 days, which is a shorter period than is recommended for patients considered high risk. The majority of events (56.6%) occurred after discharge, peaking on the eighth day. However, the study did not analyze the relationship between mobility status and risk of VTE development. A subanalysis as part of the MEDENOX study^18^ analyzed the effect of thromboprophylaxis and of mobility while in hospital, which was defined as the ability to walk more than 10 meters unaided on 10±4 days. Although the basic VTE rates were lower among patients who could walk, when compared with those who were immobile (10.6% vs. 19.7%; p = 0.03), administration of 40 mg/day of enoxaparin significantly reduced the VTE risk of patients who were mobile early, compared with placebo (3.3% vs. 10.6%; RR = 0.31; p = 0.008) and also of patients who were immobile (9.0% vs. 19.7%; RR = 0.46; p = 0.02), with no differences related to major bleeding. Thromboprophylaxis was administered for 7.3 and 7.7 days to the group who were mobilized (a mean of 4.4 days after admission) and those who were not, respectively. This study found evidence that clinical patients with initial immobility who walked early were still at risk of thrombotic events and that this risk was reduced by administration of enoxaparin at 40 mg/day.^7^^,^ 26

In current practice, many clinical patients at risk of VTE who begin to walk have their pharmacological prophylaxis withdrawn at discharge and do not complete the minimum period considered effective in clinical studies.^7^ Therefore, mobilization should not be a reason for early withdrawal of prophylaxis and each patient’s risk factors should be analyzed individually. It is important to point out that, in the above study, the subset that achieved early mobilization and still exhibited additional benefits from use of enoxaparin was at high risk of VTE: advanced age (mean of 72 years), more than two VTE risk factors (65%), respiratory failure (53%), infection (50%), NYHA III heart failure (30%), cancer (9%), and history of previous VTE (8%). Another relevant point is that, although early mobilization was defined as the ability to walk more than ten meters per day unaided, the study does not provide the mean distance actually walked per patient. There is therefore no basis on which to determine whether early mobilization, walking longer distances with elderly patients at high risk of VTE or with younger people with fewer risk factors could reduce the incidence of events, requiring a shorter duration of prophylaxis. The ideal period of pharmacological prophylaxis for clinical patients who manage to walk early on during their hospital stay remains undefined.

Another very important element is that high risk patients can still be at risk of VTE for up to 100 days after their hospital discharge.^27^ Strategies to reduce late events are dependent on identification of populations who will potentially benefit from prolonged pharmacological prophylaxis. The ideal duration of extended pharmacological prophylaxis is also unknown in these situations. The EXCLAIM study was the first to conduct a systematic analysis of immobility level and VTE risk, identifying patients who would benefit from prophylaxis with enoxaparin for a period of 28±4 days on the basis of different levels of immobility: level 1 – absolute bed rest or sedentary without bathroom privileges; level 2- total bed rest or sedentary with bathroom privileges.^8^ The benefit of extending prophylaxis was limited to patients who were female, over the age of 75 years, and classified as immobility level 1, although there was a greater risk of major bleeding when compared to placebo (0.8% vs. 0.3%; 0.51 [95% confidence interval, 0.12-0.89]). Studies of extended prophylaxis using direct action oral anticoagulants such as rivaroxaban^28^ and apixaban^29^ observed higher rates of bleeding. More recently, the Acute medically Ill venous thromboembolism Prevention with EXtended duration betrixaban (APEX)^30^ and Medically ill patient Assessment of Rivaroxaban vs. placebo IN reducing post-discharge venous thrombo-Embolism Risk (MARINER) studies^31^^,^ 32 included baseline D dimer levels in their evaluations of duration of pharmacological prophylaxis after discharge (35 to 42 days, and 45 days, respectively). In the APEX study, acutely ill clinical patients with elevated D dimer levels who had been given betrixaban did not exhibit a significant difference compared with the standard regimen with enoxaparin in terms of the pre-specified primary efficacy outcome.^30^ The MARINER study did not demonstrate a benefit from use of 10 mg rivaroxaban vs. placebo in the highest risk clinical patients for 35 days after discharge, in terms of the composite outcome of fatal or symptomatic VTE.^31^^,^ 32 Therefore, the subset of clinical patients at higher risk of VTE after discharge that would benefit from extended prophylaxis has not been identified and neither has the ideal treatment strategy.

## BENEFITS OF EARLY MOBILIZATION

While immobility can lead to a series of intercurrent medial conditions, early mobilization contributes to preventing functional deterioration and the complications associated with longer hospital stays. Early mobilization protocols are associated with better outcomes in terms of DVT incidence, shorter length of hospital stay among patients with community acquired pneumonia, improvement or maintenance of functional status of elderly hospitalized patients, and better postoperative recovery after major surgery.^33^^-^ 35 Guidelines for VTE prevention emphasize early mobilization as one of the most important components of prophylaxis and as the only prophylactic measure necessary in patients at low risk of VTE.^3^^,^ 24

## CONCLUSIONS

Current evidence is insufficient to categorically support the claim that early mobilization, in isolation, reduces the risk of events in clinical patients at high risk of VTE. Nevertheless, taking into consideration the fact that acquired restricted mobility is an independent risk factor for VTE, it is of fundamental importance that institutions implement programs to encourage early mobilization and walking. This practice does not only lead to benefits during the hospital stay, but also prepares patients for post-discharge recovery. It is very important that the multidisciplinary treating team work together in an integrated and dynamic manner to define patients’ mobility status at admission, to flag up changes as the hospital stay progresses, and to implement the interventions needed, in order to achieve a precise definition of each patient’s pharmacological prophylaxis needs. Although it can be stated that immobility increases VTE risk, to date there are no quantitative definitions for the measurements of distance or duration of ambulation that are independently correlated with reduction of VTE risk.
