# Supplementary material for: Role of ambulation to prevent venous thromboembolism in medical patients: where do we stand?
Source: J Vasc Bras. 2019 Jun 25;18:e20180107. doi: 10.1590/1677-5449.180107 (PMC6636813; doi:10.1590/1677-5449.180107)
Supplement: Translation

# Translation: Papel da deambulação na prevenção do tromboembolismo venoso em pacientes clínicos: onde estamos?

**Authors:** Maria Chiara Chindamo, Marcos Arêas Marques

**Affiliations:** 1 Universidade Federal do Rio de Janeiro – UFRJ, Departamento de Clínica Médica, Rio de Janeiro, RJ, Brasil.; 2 Rede D’Or São Luiz, Hospital Barra D’Or, Serviço de Clínica Médica, Rio de Janeiro, RJ, Brasil.; 3 Universidade do Estado do Rio de Janeiro – UERJ, Hospital Universitário Pedro Ernesto, Rio de Janeiro, RJ, Brasil.; 4 Universidade Federal do Estado do Rio de Janeiro – UNIRIO, Hospital Universitário Gaffrée e Guinle, Rio de Janeiro, RJ, Brasil.

**Keywords:** trombose venosa profunda, embolia pulmonar, tromboprofilaxia, segurança do paciente, limitação da mobilidade

## Abstract

O tromboembolismo venoso (TEV), que compreende o espectro de manifestações de trombose venosa profunda e/ou embolia pulmonar, é uma complicação comum, grave e evitável em pacientes hospitalizados. Embora a perda da mobilidade recente ou continuada represente um dos principais fatores de risco relacionados ao desenvolvimento de TEV, não existem critérios claros e uniformes para a definição do conceito de imobilidade. A diversidade dessas descrições dificulta a interpretação e a comparação dos resultados de estudos clínicos randomizados no que se refere à influência dos diferentes níveis de imobilidade na magnitude do risco de TEV e ao papel da deambulação precoce, de forma isolada, na prevenção de tais eventos. O entendimento dessas limitações é mandatório para a utilização e interpretação adequadas das ferramentas de avaliação de risco de TEV, e para a indicação da melhor estratégia de prevenção de trombose em pacientes clínicos hospitalizados. O objetivo deste estudo é revisar as principais evidências da literatura quanto ao papel da deambulação na prevenção do TEV.

## INTRODUÇÃO

O tromboembolismo venoso (TEV) representa a principal causa de morte evitável em pacientes hospitalizados^1^^,^ 2. Fatores de risco adquiridos ou hereditários estão envolvidos em sua gênese e incluem obesidade, trombose prévia, trombofilias, câncer, história recente de trauma ou de cirurgia, infarto agudo do miocárdio, acidente vascular cerebral, paresia ou paralisia de membros inferiores, idade avançada, insuficiência cardíaca congestiva, infecção aguda, terapia hormonal, cateter venoso central e admissão em unidades de terapia intensiva^3^^-^ 6. A perda da mobilidade, recente ou continuada, é um fator de risco adquirido comum e importante no desenvolvimento de TEV, aumentando a sua incidência entre duas a cinco vezes quando comparada à incidência dos pacientes com deambulação normal^5^.

Os pacientes clínicos usualmente têm histórico de redução de mobilidade devido à doença aguda que motivou a internação e à presença de comorbidades. Embora a imobilidade represente um fator de risco para indicação de profilaxia do TEV em pacientes clínicos internados, a profilaxia farmacológica ainda é subutilizada, e a deambulação é frequentemente considerada como único critério para descontinuação da tromboprofilaxia^7^. Algumas questões permanecem indefinidas e podem dificultar a avaliação precisa do risco de TEV, destacando-se: falta de consenso nas definições de imobilidade utilizadas nos grandes estudos de tromboprofilaxia em pacientes clínicos, grau de contribuição da imobilidade no risco de TEV e importância da deambulação precoce como fator de proteção. O objetivo deste estudo é revisar e avaliar as principais evidências da literatura médica quanto ao papel da deambulação na profilaxia do TEV.

## REDUÇÃO DE MOBILIDADE E RISCO DE TEV

Uma relação entre redução de mobilidade e risco aumentado de TEV, proporcional ao nível e ao tempo de restrição do paciente ao leito, foi sugerida por vários autores^8^^-^ 10. Em estudo multicêntrico caso-controle prospectivo de pacientes com idade maior que 65 anos hospitalizados, a redução da mobilidade representou um fator de risco independente de TEV (*odds ratio*: 1,73-5,64)^5^. O risco foi maior naqueles pacientes com limitação mais severa de mobilidade (repouso no leito *versus* cadeira de rodas) e com perda de mobilidade recente (< 15 dias *versus* ≥ 30 dias). Da mesma forma, a hospitalização prolongada foi considerada um fator de risco independente para o desenvolvimento de TEV, aumentando em até oito vezes a ocorrência de eventos em comparação aos pacientes não hospitalizados^11^.

## DEFINIÇÕES DE IMOBILIDADE

Diferentes definições foram empregadas em estudos clínicos randomizados para a caracterização da imobilidade: 1) qualitativas, descritas como variável dicotômica (móvel ou restrito), ou em níveis (parcial ou total); 2) quantitativas, com descrições do tempo de deambulação ou distância percorrida em 24 horas^12^^,^ 13. São utilizados como sinônimos de imobilidade os termos mobilidade reduzida, imobilidade prolongada, confinamento ao leito ou repouso com privilégio de ir ao banheiro^12^^,^ 13.

Recentemente, Ye et al.^12^ publicaram uma revisão sistemática de 21 estudos clínicos randomizados ressaltando a grande heterogeneidade na caracterização de imobilidade aplicada a pacientes clínicos hospitalizados. Algumas definições agregaram o tipo e a duração da imobilidade, como nos estudos PRIME^14^ (imobilização esperada por mais da metade do dia por período de 7 dias), PRINCE^15^ (confinamento no leito por mais de 2/3 de cada dia por 10±2 dias), ARTEMIS^16^ (confinamento ao leito ≥ 4 dias), PREVENT^17^ (hospitalização projetada por ≥ 4 dias e ≤ 3 dias de imobilização antes da hospitalização) e *Extended Clinical Prophylaxis in Acutely Ill Medical Patients* (EXCLAIM)^8^ (classificação da imobilidade em dois níveis: nível 1, repouso absoluto ou sedentário sem privilégio de ir ao banheiro e nível 2, repouso com privilégio de ir ao banheiro, considerando ≥ 3 dias previstos de hospitalização). O estudo *Prophylaxis of Venous Thromboembolism in MEDical Patients With ENOXaparin* (MEDENOX)^18^ utilizou parâmetros quantitativos, definindo imobilidade como incapacidade de atingir distância de caminhada autônoma > 10 metros no período de 10±4 dias.

Nesta revisão, os autores concluíram que, apesar da eficácia estabelecida da farmacoprofilaxia do TEV em pacientes portadores de doença clínica aguda, ainda não há consenso sobre a definição de imobilidade^12^. A diversidade de definições e as descrições predominantemente qualitativas dificultam a comparação de resultados e impossibilitam determinar, de forma precisa, níveis de mobilização/deambulação que possam contribuir para a redução da incidência de eventos de TEV^19^^,^ 20.

## IMOBILIDADE E MODELOS DE ESTRATIFICAÇÃO DE RISCO DE TEV EM PACIENTES CLÍNICOS

Ainda não foi definido o modelo mais adequado de estratificação de risco de TEV em pacientes clínicos agudamente doentes^21^^-^ 23. Essas ferramentas são muito importantes na identificação dos pacientes elegíveis para profilaxia farmacológica ou mecânica durante a hospitalização. Uma vez que protocolos de tromboprofilaxia são pouco implementados em todo mundo, diversos modelos de estratificação de risco de TEV foram desenvolvidos para embasar a decisão clínica, melhorando o uso da profilaxia medicamentosa na população de risco^21^^-^ 23.

Todos os modelos de estratificação validados incluem a redução da mobilidade como fator de risco para o TEV. Em sua última atualização, a diretriz do *American College of Chest Physicians* (ACCP)^3^ recomenda o uso do escore de Padua como ferramenta de avaliação do risco de TEV em pacientes clínicos hospitalizados. Este consiste em um modelo composto de 11 fatores de risco, pontuados de um a três (escore máximo de 20 pontos), para identificar pacientes clínicos de alto risco de TEV (escore ≥ 4)^22^. Nesse escore, a redução de mobilidade já confere três pontos. Outro modelo quantitativo também validado externamente é o *International Medical Prevention Registry on Venous Thromboembolism* (IMPROVE), que apresenta uma definição mais abrangente de imobilidade^23^. No Brasil, um grupo de especialistas publicou em 2007 a Diretriz Brasileira de Profilaxia de TEV em Paciente Clínico Internado, que precedeu a publicação dos escores acima descritos. Nessa diretriz, idade superior a 40 anos e imobilidade, associadas à presença de pelo menos um fator de risco para TEV, indicam a utilização de farmacoprofilaxia^24^. As definições de imobilidade desses três modelos de avaliação de risco estão discriminadas na Tabela 1.

**Tabela 1:** Definição de imobilidade nos modelos de avaliação de risco de TEV.

**Modelos de avaliação de risco**	**Definição de imobilidade**	**Indicação de profilaxia farmacológica**
Escore de Padua^22^	Probabilidade de imobilidade por limitações causadas por doença ou tratamento ou por indicação médica durante pelo menos 3 dias	Imobilidade: 3 pontos. Benefício de profilaxia farmacológica: escore ≥ 4
IMPROVE^23^	Confinamento em leito ou cadeira > 24 horas por ≥ 7 dias	Imobilidade: 1 ponto. Benefício de profilaxia farmacológica: escore ≥ 2
Diretriz Brasileira de Profilaxia em Paciente Clínico Internado^24^	Devido à doença passa pelo menos a metade do dia deitado ou sentado na beira do leito (excluindo-se o período de sono)	Profilaxia indicada em caso de imobilidade e idade ≥ 40 anos + pelo menos um fator de risco

IMPROVE = *International Medical Prevention Registry on Venous Thromboembolism*.

## IMOBILIDADE E DETERMINAÇÃO DO TEMPO DE PROFILAXIA

Definir a relação entre a duração do risco de TEV, tempo de utilização da farmacoprofilaxia e deambulação precoce em pacientes clínicos hospitalizados permanece um desafio na prática clínica. Além das conhecidas limitações relacionadas às diversas definições de imobilidade, também não existem padrões mensuráveis de deambulação que se correlacionem com a redução do risco de TEV.

Embora o tempo estabelecido para profilaxia eficaz e segura em pacientes clínicos hospitalizados seja de 6 a 14 dias (média de 7 dias), conforme definição dos estudos PREVENT, ARTEMIS e MEDENOX^16^^-^ 18, a deambulação tem sido frequentemente utilizada como critério único para descontinuação da profilaxia^7^. Considerando a tendência universal de diminuição do tempo de permanência hospitalar, torna-se improvável, na prática atual, que pacientes clínicos recebam a farmacoprofilaxia pela duração preconizada enquanto internados. Menos de 2% dos pacientes continuam a receber farmacoprofilaxia após a alta hospitalar, o que provoca um aumento da incidência de eventos fora do hospital^25^^,^ 26.

Em estudo de vida real, Amin et al.^26^ avaliaram retrospectivamente a ocorrência de TEV sintomático em até 180 dias após admissão em 11.139 pacientes com diagnóstico de câncer, insuficiência cardíaca, doença pulmonar grave ou doença infecciosa. A taxa de TEV foi de 3,3% após receber farmacoprofilaxia em média por 5 dias, período inferior ao recomendado para pacientes considerados de alto risco. A maioria dos eventos (56,6%) ocorreu após a alta, com o pico no oitavo dia; no entanto, não foi estudada a relação do *status* de mobilidade com o risco de desenvolvimento do TEV. Em subanálise do estudo MEDENOX^18^, foi analisado o efeito da tromboprofilaxia e da deambulação durante a internação, definida como a capacidade de atingir uma distância de caminhada autônoma superior a dez metros em 10±4 dias. Embora as taxas basais de TEV tenham sido menores em pacientes que deambularam, quando comparadas aos que não deambularam (10,6% *versus* 19,7%; p = 0,03), a administração de enoxaparina 40 mg/dia reduziu significativamente o risco de TEV nos pacientes que caminharam precocemente em comparação ao uso de placebo (3,3% *versus* 10,6%; RR = 0,31; p = 0,008), como também nos pacientes com imobilidade (9,0% *versus* 19,7%; RR = 0,46; p = 0,02), sem diferenças relacionadas a sangramento maior. A tromboprofilaxia foi fornecida respectivamente por 7,3 e 7,7 dias no grupo que passou a deambular (em média 4,4 dias após a admissão) e nos que não deambularam. Esse estudo forneceu evidências de que pacientes clínicos com imobilidade inicial e que passaram a deambular precocemente ainda permaneceram sob risco de eventos de trombose, que foi reduzido com o uso de enoxaparina 40 mg/dia^7^^,^ 26.

Na prática atual, muitos pacientes clínicos sob risco de TEV que começam a deambular têm a farmacoprofilaxia suspensa na alta e não completam o período mínimo considerado eficaz nos estudos clínicos^7^. Dessa forma, a deambulação não deve ser motivo para interromper precocemente a profilaxia, devendo-se considerar os fatores de risco individuais de cada paciente. É importante ressaltar que, no estudo acima, a população que deambulou e apresentou benefício adicional com uso de enoxaparina era de alto risco de TEV: idade avançada (média 72 anos), presença de mais de dois fatores de risco para TEV (65%), insuficiência respiratória (53%), infecção (50%), insuficiência cardíaca NYHA III (30%), câncer (9%) e história de TEV prévio (8%). Outro ponto relevante é que, apesar da deambulação precoce ter sido definida como a capacidade de atingir caminhada autônoma superior a dez metros por dia, não há referência no estudo sobre a distância média percorrida por paciente. Portanto, não existem evidências para determinar se a deambulação precoce por distâncias maiores em idosos com alto risco para TEV, ou em indivíduos jovens com menos fatores de risco, poderia reduzir a incidência de eventos, implicando em um tempo menor de profilaxia. O tempo ideal de farmacoprofilaxia nos pacientes clínicos que deambulam precocemente durante a internação ainda permanece indefinido.

Outro aspecto fundamental é que o risco de TEV pode se manter por até 100 dias após a hospitalização nos pacientes de alto risco^27^. Estratégias para redução dos eventos tardios dependem do reconhecimento das populações com potencial benefício da farmacoprofilaxia por tempo prolongado. A duração ideal da profilaxia farmacológica estendida nesse contexto ainda é incerta. O estudo EXCLAIM foi o primeiro a avaliar sistematicamente o nível de imobilidade e o risco de TEV, identificando pacientes que se beneficiariam da profilaxia com enoxaparina por período de 28±4 dias considerando níveis distintos de imobilidade: nível 1- repouso absoluto ou sedentário sem privilégios de ir ao banheiro; nível 2- repouso total ou sedentário com privilégios de ir ao banheiro^8^. O benefício da extensão da profilaxia foi limitado a pacientes do sexo feminino com idade superior a 75 anos e imobilidade nível 1, incorrendo, no entanto, em risco de sangramento maior quando comparado ao placebo (0,8% *versus* 0,3%; 0,51 [IC95%, 0,12-0,89]). Estudos de profilaxia estendida utilizando anticoagulantes orais de ação direta como rivaroxabana^28^ e apixabana^29^ demonstraram maiores taxas de sangramento. Mais recentemente, os estudos *Acute medically Ill venous thromboembolism Prevention with EXtended duration betrixaban* (APEX) (betrixabana)^30^ e *Medically ill patient Assessment of Rivaroxaban versus placebo IN reducing post-discharge venous thrombo-Embolism Risk* (MARINER) (rivaroxabana)^31^^,^ 32 incorporaram a dosagem do d-dímero basal na avaliação da extensão da farmacoprofilaxia após a alta (35 a 42 dias e 45 dias, respectivamente). No estudo APEX, pacientes clínicos agudamente doentes com valor elevado de d-dímero que utilizaram betrixabana não apresentaram diferença significativa comparada ao regime padrão com enoxaparina no resultado primário de eficácia pré-especificado^30^. O estudo MARINER não demonstrou benefício do uso de rivaroxabana 10 mg *versus* placebo em pacientes clínicos de mais alto risco por 35 dias após alta, relacionado ao desfecho composto de TEV fatal ou sintomático^31^^,^ 32. Portanto, não está ainda estabelecido o subgrupo de pacientes clínicos sob maior risco de TEV após a alta que possa se beneficiar da profilaxia estendida, nem a estratégia terapêutica mais adequada.

## BENEFÍCIOS DA MOBILIZAÇÃO PRECOCE

Enquanto a imobilidade pode levar a uma série de intercorrências médicas, a mobilização precoce contribui para a prevenção do declínio funcional e de complicações associadas a um maior período de hospitalização. Protocolos de mobilização precoce estão associados a melhores desfechos quanto à incidência de TVP, menor tempo de internação de pacientes com pneumonia adquirida na comunidade, melhora ou manutenção do estado funcional na alta de pacientes idosos hospitalizados e melhor recuperação em pós-operatório de grandes cirurgias^33^^-^ 35. As diretrizes de prevenção de TEV enfatizam a mobilização precoce como um dos componentes principais da profilaxia e como a única medida profilática necessária em pacientes de baixo risco para TEV^3^^,^ 24.

## CONCLUSÃO

As evidências atuais não permitem afirmar de forma categórica que a deambulação precoce, isoladamente, reduz o risco de eventos em pacientes clínicos com alto risco para o desenvolvimento do TEV. No entanto, levando em consideração que a redução adquirida da mobilidade constitui um fator de risco independente para TEV, é fundamental que as instituições estabeleçam programas de incentivo à mobilização e à deambulação precoce dos pacientes. Essa prática traz benefícios não só durante a hospitalização, mas também prepara o paciente para a recuperação após a alta. O trabalho integrado e dinâmico da equipe multidisciplinar para definir o *status* de mobilidade do paciente na admissão, sinalizar mudanças ao longo da hospitalização e realizar as intervenções necessárias é fundamental para a definição precisa da necessidade de profilaxia farmacológica. Embora possamos afirmar que a imobilidade aumenta o risco de TEV, não existem até o momento definições quantitativas de medidas de distância ou tempo de deambulação que se correlacionem de forma independente com a redução do risco de TEV.
